# Possible Pathogenesis and Prevention of Long COVID: SARS-CoV-2-Induced Mitochondrial Disorder

**DOI:** 10.3390/ijms24098034

**Published:** 2023-04-28

**Authors:** Tsung-Hsien Chen, Chia-Jung Chang, Peir-Haur Hung

**Affiliations:** 1 Department of Internal Medicine, Ditmanson Medical Foundation Chia-Yi Christian Hospital, Chiayi 60002, Taiwan; cych13794@gmail.com; 2 Division of Critical Care Medicine, Department of Internal Medicine, Ditmanson Medical Foundation Chia-Yi Christian Hospital, Chiayi 60002, Taiwan; 3 Department of Life and Health Science, Chia-Nan University of Pharmacy and Science, Tainan 717301, Taiwan

**Keywords:** SARS-CoV-2, long COVID, pathogen-associated molecular patterns (PAMPs), mitochondrial disorder, inflammatory responses, oxidative phosphorylation, electron transport chain

## Abstract

Patients who have recovered from coronavirus disease 2019 (COVID-19) infection may experience chronic fatigue when exercising, despite no obvious heart or lung abnormalities. The present lack of effective treatments makes managing long COVID a major challenge. One of the underlying mechanisms of long COVID may be mitochondrial dysfunction. Severe acute respiratory syndrome coronavirus 2 (SARS-CoV-2) infections can alter the mitochondria responsible for energy production in cells. This alteration leads to mitochondrial dysfunction which, in turn, increases oxidative stress. Ultimately, this results in a loss of mitochondrial integrity and cell death. Moreover, viral proteins can bind to mitochondrial complexes, disrupting mitochondrial function and causing the immune cells to over-react. This over-reaction leads to inflammation and potentially long COVID symptoms. It is important to note that the roles of mitochondrial damage and inflammatory responses caused by SARS-CoV-2 in the development of long COVID are still being elucidated. Targeting mitochondrial function may provide promising new clinical approaches for long-COVID patients; however, further studies are needed to evaluate the safety and efficacy of such approaches.

## 1. Introduction

The primary symptom of coronavirus disease 2019 (COVID-19) is a respiratory illness [1]. However, severe acute respiratory syndrome coronavirus 2 (SARS-CoV-2) can also cause gastrointestinal symptoms and inflammation, which can affect the lungs and immune response through cytokines in the bloodstream [2]. People with post-COVID-19 illness may experience a variety of symptoms that can persist for weeks, months, or even years after infection [3]. Sometimes, symptoms even disappear and reappear. These symptoms can be difficult to explain and manage. Long COVID has been defined as “signs, symptoms, and conditions that continue or develop after initial SARS-CoV-2 infection” [4]. Clinical evaluations and the assessment of results of routine blood work, chest X-rays, and electrocardiograms may be normal [5]. The most commonly experienced symptoms of post-COVID-19 conditions include an exaggerated hyperventilation response during exercise, tiredness or weakness that interferes with daily life, malaise after exertion, fever, and more [6,7]. The peak oxygen uptake (VO_2peak_) in patients with long COVID-19 has been found to be significantly lower than normal [8]. In the first few months after infection, due to chronotropic insufficiency and reduced cardiac output, the cardiovascular system can lead to low VO_2peak_ through lower- than-normal cardiac output [8]. Alternatively, peripheral factors such as muscle mass, strength and perfusion, mitochondrial function, or arteriovenous oxygen difference may also contribute to low VO_2peak_ [8]. These symptoms are similar to those reported by patients with myalgic encephalomyelitis/chronic fatigue syndrome and people with genetic mitochondrial diseases [7,9].

Co-localization of the SARS-CoV-2 genome and mitochondria in SARS-CoV-2-infected tissues has been reported [10]. Mitochondria can be extremely vulnerable to physiological and pathological stimuli such as viral infections. The integrity and normal function of mitochondria can be altered during SARS-CoV-2 infection, thereby modulating cellular responses. The replication of SARS-CoV-2 begins with virus-induced double-membrane vesicles (DMV) that originate from the endoplasmic reticulum and eventually integrate to form a complex membrane network [11]. SARS-CoV-2 proteins are known to selectively target organelle compartments such as the endoplasmic reticulum and mitochondria, and they can reside in the host mitochondrial matrix [12]. This results in mitochondrial dysfunction and increased oxidative stress, ultimately leading to the loss of mitochondrial integrity and cell death [13]. These proteins also combine with non-specific and selective channel mitochondrial permeability transition pore (mPTP) complexes on mitochondria, which could alter the mitochondrial morphology and function, disrupt the Ca^2+^ cycle, inhibit voltage-gated calcium channel (VDCC) activity, and lead to ion homeostasis disorder and mitochondrial dysfunction, thereby disrupting the cellular Ca^2+^ cycle and cell viability [14].

Furthermore, hyperactivation of the NLR family pyrin domain-containing 3 (NLRP3) inflammasome results in hyper-responsiveness in classically activated macrophages when mitochondrial function is compromised, increasing mitochondrial reactive oxygen species (mtROS), and free circulating mitochondrial DNA (mtDNA). Oxidized mtDNA, cardiolipin, and cytochrome c from the damaged mitochondria, when released into the cytoplasm, activate damage-associated molecular patterns (DAMPs), leading to sustained local and systemic inflammatory responses [15,16,17]. This article reviews hypotheses regarding the changes in mitochondrial status under SARS-CoV-2 infection, including mitochondrial redox homeostasis, regulation of inflammation, and antiviral signaling, among others, and discusses why mitochondrial damage may contribute to long COVID.

## 2. SARS-CoV-2 and Long COVID

### 2.1. SARS-CoV-2 Non-Structural Proteins

Coronavirus is a single-stranded positive-sense RNA virus containing an envelope. Its genome is structured as 5′-leader-UTR-replicase (open reading frame 1ab)-spike-envelope-membrane-nucleocapsid-3′UTR-polyA tail [18,19]. SARS-CoV-2 virus particles are composed of main structural proteins, such as the spike protein, envelope protein, membrane protein, and nucleocapsid protein. The replicase polyproteins self-cleave the ORF1a and ORF1ab polyproteins of SARS-CoV-2 to form 16 non-structural proteins (NSP1 to NSP16) [20]. These complexes are involved in replication and transcription, playing different roles throughout the coronavirus life cycle (Table 1). For example, NSP16 plays an important role in evading the host immune response and protecting the nascent viral mRNA from degradation [21]. 

In addition to these non-structural protein complexes involved in the replication and transcription of SARS, the open reading frame 3a (ORF3a), ORF3b, ORF3c, ORF3d, ORF6, ORF7a, ORF7b, ORF8, ORF9b, ORF9c, and ORF10 are also involved in the infection mechanism of SARS-CoV-2 (Table 2) [22]. In particular, SARS-CoV-2 lacks ORF8a, compared to SARS-CoV [23,24].

### 2.2. Viral PAMPs

**Figure 1:**
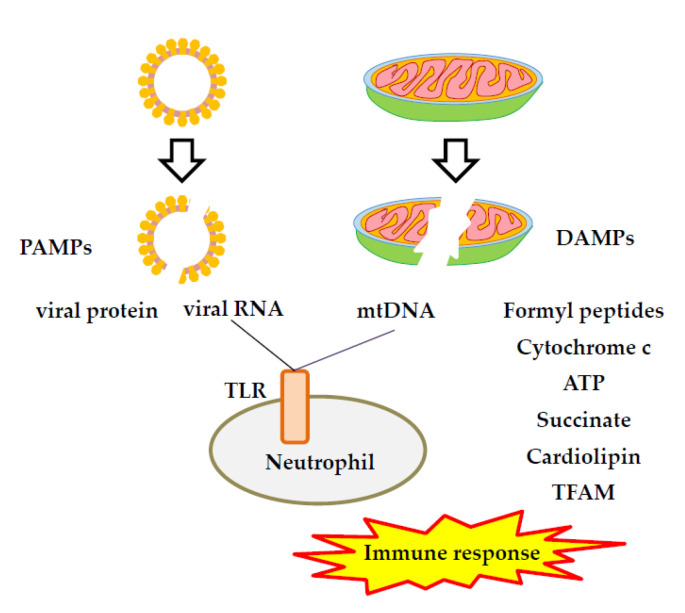
Mitochondrial damage-associated molecular patterns (DAMPs) and related pathogen-associated molecular patterns (PAMPs) in the chronic inflammasome. Inflammation is caused by damage or infection by stimulatory signals, including PAMPs and DAMPs. PAMPs are derived from microbial products such as viral nucleic acids, which trigger inflammation in response to infection. DAMPs originate from host cells or products released by cells in response to signals such as hypoxia, producing sterile inflammatory responses in settings such as myocardial infarction, cancer, autoimmune disease, and atherosclerosis. ATP, adenosine triphosphate; DAMPs, damage-associated molecular patterns; mtDNA, mitochondrial DNA; TFAM, transcription factor A mitochondria; TLR, toll-like receptor.

When the immune system senses tissue damage, certain molecules are released to trigger an immune response via DAMPs [53]. DAMPs and related pathogen-associated molecular patterns (PAMPs) trigger inflammasome assembly, activate caspase-1 [54], and induce mitochondrial damage [55] (Figure 1). Toll-like receptors (TLRs) are expressed on both innate and non-immune cells. The activation of TLRs plays a dual role [56], including the recognition and elimination of PAMPs in bacteria, viruses, and other pathogens, and the involvement of pathogens in the recognition and elimination of autogenous DAMPs released from dying or lysed cells [57]. Typical PAMPs are nucleic acids, including viral RNA and DNA, but also include surface-exposed glycoproteins, lipoproteins, and various membrane components [57]. The engagement of PAMPs and/or DAMPs can activate pattern recognition receptors (PRRs), such as TLRs or nucleotide-binding oligomerization domain-containing protein 2 (NOD2), resulting in NFκB activation and the activation of gene transcription. It was earlier reported that double-stranded RNA (dsRNA) viral PAMPs are known to activate TLR3 [58], whereas single-stranded RNA (ssRNA) viral PAMPs mainly activate TLR7/8 [59]. Changes in the distribution of charged amino acids in the spike protein of Omicron variants interfere with the recognition of TLRs by PRRs, thereby reducing activation of the NF-κB pathway and related signaling pathways, consequently inhibiting viral replication and systemic immune hyperactivation [60].

### 2.3. Long COVID

“Post-COVID-19 syndrome”, “long COVID-19”, “long-term COVID-19 effects”, “long haulers”, “COVID-19 long tail”, and “persistent COVID-19 symptoms” [61,62,63,64] are all terms used to describe various conditions, such as inflammation, sequelae of organ damage, hospitalization, and social isolation, which persist for a long time after SARS-CoV-2 infection [65]. Long COVID has long-term heterogeneity and complex symptoms [66]. The most common symptoms include chronic fatigue [67]; respiratory manifestations (e.g., cough and shortness of breath) [67]; arrhythmias, palpitations, hypotension, increased heart rate, venous thromboembolic disease, myocarditis, and acute heart failure [68]; and symptoms of neurological and psychiatric related disorders (e.g., peripheral neuropathy, brain fog, anxiety, and depression) [67,69,70]. Diagnostic clusters have shown that long COVID does not have a single phenotype but, instead, a collection of sub-phenotypes subject to different diagnoses and treatments [71].

The mechanism underlying the persistence of long COVID has not yet been identified [72], but it has been speculated that it may be related to abnormal immune responses, virus-specific pathophysiology, inflammatory damage in response to acute infection [73], persistence of the virus in certain tissues [74,75] or exosomes and hypertrophy cells [76,77], SARS-CoV-2 interactions with the host microbiome/virome community, coagulation/coagulation problems, and dysfunctional brainstem/vagal signaling [78]. In addition, the role of SARS-CoV-2 in relation to mitochondrial damage and the subsequent immune response has been recently considered [79,80,81]. For example, the spike protein of SARS-CoV-2 can inhibit the transcription of mitochondrial metabolic genes in the long term, resulting in myocardial fibrosis and myocardial contractile dysfunction [79]. Long COVID neurological symptoms are associated with SARS-CoV-2 proteins and abnormalities in mitochondrial proteins in nerve cells [81].

## 3. Altered Mitochondrial Function in SARS-CoV-2 Infection

Both SARS-CoV-2-induced and inactivity-induced changes may increase mitochondrial dysfunction and myofibril breakdown, and decrease mitochondrial biogenesis and muscle synthesis [82]. Cells infected with SARS-CoV-2 exhibit abnormally swollen mitochondrial cristae [83]. Mitochondria in SARS-CoV-2-infected cells are markedly thinned, and the mitochondria are translocated and clustered around viral dsRNA containing DMV [84]. NSP4 and ORF9b cause structural changes in mitochondria, as well as the formation of outer membrane macropores and release of mtDNA-laden inner membrane vesicles [85]. In addition, genes related to mitochondria are predominantly downregulated in SARS-CoV-2-infected cells [86]; for example, the expression of mtDNA, antioxidants (e.g., catalase, glutathione synthetase), and mitochondrial respiratory chain proteins (NDUFA9, NADH: ubiquinone oxidoreductase subunit A9; SDHA, succinate dehydrogenase complex flavoprotein subunit A; COX4I1, cytochrome c oxidase subunit 4I1) were significantly reduced in SARS-CoV-2-infected placentas [87]. 

SARS-CoV ORF7a, ORF8a, and ORF9b are known to be located in the mitochondria, which can inhibit retinoic acid-inducible gene I-mitochondrial antiviral signaling protein (RIG1-MAVS)-dependent interferon signaling, enhance viral replication, and destroy mitochondrial function [88]. Mitochondrial dysfunction is one of the earliest and most prominent neurodegenerative features of SARS-CoV-2-induced neuropathology [89]. In addition, metabolic syndromes such as diabetes, obesity, and cardiovascular and liver diseases —which are related to mitochondria—also lead to susceptibility and adverse outcomes of SARS-CoV-2 infection [90,91,92,93], significantly increasing the mortality rate of SARS-CoV-2 infection [94,95]. Many environmental chemicals, malnutrition, and exacerbated socio-economic stress can also cause mitochondrial damage, which can negatively affect the prognosis of COVID-19 [96]. Furthermore, cardiac mitochondrial disruption after infection, ROS production, and energetic stress lead to mitochondrial alterations and cardiovascular dysfunction in COVID-19 patients, and the incidence of adverse cardiovascular events increases in recovered COVID-19 patients [97].

### 3.1. Alteration of Mitochondrial Ca^2+^ Signaling

The host Ca^2+^ channel affects every process of infection by the virus, which invades the host Ca^2+^ channel to disrupt homeostasis and benefit its lifecycle at the expense of host survival. When inhibiting the activity of store-operated Ca^2+^ channels, viral shedding is reduced, thereby reducing the infectivity of many enveloped RNA viruses [98,99]. During viral infection, infectious agents are also known to trigger dysregulation of host cell signaling cascades that affect cellular calcium dynamics [100,101]. This can lead to an imbalance in calcium concentrations, which can have an impact on mitochondrial function. Furthermore, six specific acidic residues (E819, D820, D830, D839, D843, and D848) on the SARS-CoV-2 spike proteins may bind calcium, thereby regulating the ability of the virus to insert into lipid membranes, which, in turn, affects oxidative phosphorylation (OXPHOS) and mitochondrial calcium sequestration [14,102,103]. 

The endoplasmic reticulum, mitochondria, and plasma membrane are all involved in the coordination of calcium dynamics, and there is evidence that viruses themselves may cause alterations in mitochondrial calcium signaling. ORF3a is known to assemble into dimeric folds and form non-selective Ca^2+^ permeable cation channels [104]. When ORF3 is expressed, the mitochondria-associated membrane (MAM) formation is increased and cytoplasmic calcium is transported to mitochondria through the endoplasmic reticulum, as ORF3a is a calcium ion transporter [104]. NSP2 and NSP4 interact with the endoplasmic reticulum lipid raft-associated protein 1/2 (ERLIN1/2) complex to regulate Ca^2+^ signaling from the endoplasmic reticulum to the mitochondria [105]. Furthermore, the calcium influx due to ORF3a activates the switch for calcium-dependent caspases and apoptosis which, in turn, trigger programmed cell death [106]. SARS-CoV-2 proteins significantly disrupt the baseline oscillations of cellular calcium, block L-type calcium channel activity, and cause cellular damage [14]. SARS-CoV-2 proteins alter mPTP, causing it to open and trigger cell death [14]. The mPTP complex components SPG7 matrix AAA peptidase subunit (SPG7) and cyclophilin D are upregulated in cells infected with SARS-CoV-2 [107]. SPG7, peptidylprolyl isomerase F (PPIF), and mitochondrial matrix import factor 23 (MIX23) are known to interact. The knockdown of MIX23 in mitochondria significantly enhanced the ability of mitochondria to sequester calcium. In contrast, an mPTP blocker cyclosporin A is known to restore mitochondrial Ca^2+^ retention and cell viability after viral infection [14]. ORF3a, ORF9b and 9c, ORF10, and NSP6 of SARS-CoV-2 are associated with mPTP complex proteins involved in mitochondrial dysfunction and cell death [14].

### 3.2. Alteration in Glycolysis–OXPHOS Equilibrium

Long COVID is a virus-induced chronic and self-perpetuating unresolved state of metabolic imbalance characterized by mitochondrial dysfunction, ROS continuously driving inflammation, and a shift towards glycolysis [108]. The functions of OXPHOS and mPTP can be altered by viruses, resulting in an imbalance of energy and biosynthetic resources or evasion of immune surveillance [109,110]. The NSP4 of SARS-CoV-2 interacts with the mitochondrial chaperone ion peptidase 1 (LONP1), affecting the function of LONP1 to promote mitochondrial protein folding, together with the mitochondrial 70 kDa heat-shock protein (mtHSP70) chaperone system [111]. OXPHOS assembled with an insufficient amount of proteins will have defects, affecting the complex function of OXPHOS. For example, in the bronchoalveolar lavage fluid of patients infected with SARS-CoV-2, the nicotinamide adenine dinucleotide (NADH):ubiquinone oxidoreductase core sub-unit V (NDUFV, complex 1), SDHA (complex 2), COR1 (cytochrome c reductase), ubiquinol–cytochrome c reductase core protein 2 (UQCRC2, complex 3), adenosine triphosphate (ATP) synthase peripheral stalk sub-unit F6 (ATP5PF), ATP synthase F1 sub-unit alpha (ATP5F1A), ATP synthase peripheral stalk sub-unit d (ATP5PD), F-type ATPase A, and inorganic pyrophosphatase 2 (PPA2, ATP synthase) were down-regulated [112]. The OXPHOS of infected patients may not function properly, leading to decreased mitochondrial aerobic respiration and higher oxidative stress. The integrity of the mitochondrial membrane may be compromised after SARS-CoV-2 infection. Complexes II and IV of OXPHOS component proteins were found outside the virus-infected cytoplasm and mitochondrial matrix, indicating that the functions of OXPHOS and the electron transport chain were disrupted [113]. An impaired expression of mitochondrial OXPHOS and antioxidant genes was observed, and the loss of electron transport chain and membrane potential resulted in decreased mitochondrial aerobic respiration and enhanced mtROS [114]. Mitochondrial damage initiates apoptosis under impaired mitochondrial electron transport chain activity, ATP depletion, and increased oxidative stress [115].

Hyperlactation is observed in all COVID-19 deaths [116]. Patients infected with SARS-CoV-2 have altered metabolic pathways that lead to decreased mitochondrial respiration and increased glycolysis, which may worsen the patient’s condition. The SARS-CoV-2 proteins responsible for exacerbation of the disease prompt the cells to switch from an oxidative to a glycolytic phenotype [14]; for example, the spike protein of SARS-CoV-2 decreases basal mitochondrial respiration and ATP production, increases glucose-induced glycolysis, and maximizes the glycolytic transport capacity of the port [117]. This implies that SARS-CoV-2 can take over host mitochondria to facilitate its metabolic pathways [48,118,119,120], resulting in higher lactate and plasma glucose values in SARS-CoV-2-infected patients. When cells were infected with SARS-CoV-2 membrane protein or ORF3a, there was a lower basal mitochondrial oxygen consumption rate (OCR) and a minimal trifluoromethoxy carbonylcyanide phenylhydrazone (FCCP)-induced maximal OCR. NSP6 and NSP7 partially inhibited the maximal OCR in infected cells [14]. Certain SARS-CoV-2 proteins cause cells to switch from an oxidative to a glycolytic phenotype [14]. Hypoxia is induced by lung involvement, favoring glycolysis and lactate accumulation when mitochondrial dysfunction and OXPHOS are reduced. The accumulation of lactate is detrimental to the function of MAVS, and may further reduce the interferon response [121]. Patients infected with SARS-CoV-2 have altered metabolic pathways that lead to decreased mitochondrial respiration and increased glycolysis, which may worsen the patient’s condition. This implies that SARS-CoV-2 can take over the host mitochondria, to obstruct its metabolic pathways. Furthermore, the lactate and plasma glucose values are higher in SARS-CoV-2 infected patients.

### 3.3. Alteration of Mitochondrial Dynamics

The fusion and fission dynamics of mitochondria remodel the ultrastructure of the membrane [122], allowing for the removal of damaged mitochondria [123]. The mitochondrial morphology is altered or destabilized in the normal physiological division–fusion dynamics of cells, which plays a role in many pathological conditions [124]. For example, inhibiting the mitochondrial fusion-essential protein mitofusin-2 (Mfn2) increased ROS production and increased c-Jun N-terminal kinase (JNK) and nuclear factor kappa-light chain enhancer (NFκB) in activated B cell signaling, resulting in decreased insulin signaling and glucose uptake [125]. Additionally, the aberrant expression of the dynein-related protein 1 (DRP1, mitochondrial fission protein) increased H_2_O_2_ production and damaged mitochondria [126].

The ORF9b protein mediates the degradation of the DRP1, leading to the abnormal lengthening of mitochondria [46]. Moreover, infected cells present a down-regulation of genes associated with mitochondrial ribosome synthesis, mitochondrial complex I synthesis, translocase, mitochondrial fission process 1 (MTFP1, mitochondrial fission-promoting protein), and suppressor of cytokine signaling 6 (SOCS6) [86]. The down-regulation of the mechanistic target of rapamycin complex 1 (mTORC1) resulted in decreased expression of MTFP1 and complex I [127,128]. Reduced expression of MTFP1 activates nuclear factor kappa-light-chain-enhancer of activated B cells (NFκB) and SOCS6, leading to mitochondrial hyper fusion [129].

### 3.4. Alteration of Autophagy and Apoptosis

Autophagy denotes the removal of unused proteins and damaged mitochondria from cells, but impaired autophagy leads to reduced oxygen consumption and reduced mitochondrial activity. Autophagy can also transform cells into pro-inflammatory generators, and lead to protein clumps that disrupt various processes. Mitochondrial outer membrane permeability (MOMP) is controlled by mitochondria [130], which triggers apoptosis during intracellular DNA damage, oxidative stress, cytosolic Ca^2+^ overload, endoplasmic reticulum stress, and other perturbations [131]. Viruses deflect mitochondrial function through pro- and anti-apoptotic responses [132]. When pro-apoptotic signals prevail, the outer membrane loses its integrity and re-releases several potentially toxic intermembrane-space (IMS) proteins into the cytoplasm. IMS are driven to release apoptosis-inducing factors in response to DNA damage which, in turn, leads to DNA damage-associated enzyme polymerization (ADP-ribose polymerase, PARP) [133,134,135]. When host cells send out PAMP signals, inflammasomes oligomerize and form receptors, activate caspases to produce cytokines, and induce apoptosis [54]. The inflammasome is activated, leading to the production of pro-inflammatory cytokines (IL-1β and 12) and apoptosis [136]. The permeabilization of mitochondria is critical for apoptosis and regulated necrosis [130,131,137]. 

ORF6 localizes to the autophagosome and lysosomal membranes [33]. Furthermore, ORF9b induces autophagy in autophagy-related 5 (ATG5)-mediated host cells [46]. ORF3a and ORF8 are involved in apoptosis [42]. NSP4 interacts with B-cell lymphoma 2 (BCL2) antagonist/killer (BAK) to mediate macropore formation ability. ORF9b inhibits the anti-apoptotic member of the BCL2 family protein myeloid leukemia-1 (MCL1) and induces the formation of inner membrane vesicles containing mtDNA. The removal of BAK and/or over-expression of MCL1 significantly reverses SARS-CoV-2-mediated mitochondrial damage. Meanwhile, ORF9b and ORF9c interact with organelles, leading to the inhibition of antiviral responses in infected cells [138].

### 3.5. Cell Death and mtDNA Release 

The activation of MOMP leads to cell death through cytochrome c release, apoptosis protease activating factor-1 (APAF-1)-dependent, and deoxyadenosine triphosphate (dATP)-dependent assembly, followed by caspase activation and the involvement of proinflammatory signaling functions. ORF3a is involved in necrotic cell death and lysosomal damage [28], and influences viral replication and virulence [26]. ORF6 and ORF7a have been shown to display the highest potency for cytotoxicity [33]. 

SARS-CoV-2 infects mitochondria and forms double-membrane vesicles which, in turn, leads to the release of mtDNA into circulation and the activation of the immune response, resulting in a severe pro-inflammatory state [139]. The mtDNA variation in COVID-19 patients can predict the degree of disease and the risk of developing severe disease [140,141]. After apoptosis, mtDNA is released, further aggravating local and systemic inflammatory responses [142]. Positive correlations between mtDNA levels and pro-inflammatory markers have been determined in the plasma of COVID-19 patients [143,144]. This is now used an indicator of COVID-19 severity or a predictor of mortality. Moreover, when mtDNA is released into the cytosol, it acts as a DAMP, which, in turn, causes a “cytokine storm” and inhibits cellular regulation of excessive inflammatory responses [143].

## 4. Mitochondrial Redox Status and Inflammatory Response

In individuals with poorly functioning mitochondria, the virus may “tip” the host into a cycle of chronic inflammation. Mitochondria play a vital role in cellular energy production and are a key component of the innate immune response. When the mitochondrial membrane and electron transport chain complex are disrupted, more ROS are generated due to the loss of membrane potential, which can lead to oxidative stress [114] and the activation of pro-inflammatory pathways [145]. While ROS production is critical in immune responses, excess ROS can damage host tissues and exacerbate inflammation [146]. Finally, it can cause damage to tissues or organs, leading to such consequences as muscle disorders [147]. In fact, mtROS —which is higher in SARS-CoV-2-infected cells and monocytes—can enhance inflammatory responses through the up-regulation of IL1-α [112,148]. Furthermore, DAMPs such as oxidized mtDNA can be released into the cytoplasm, which aggravates inflammation by activating the inflammasome [149]. When host cells send out PAMP signals, inflammasomes oligomerize and form receptors, activate caspases to produce cytokines, and induce apoptosis [54].

The ORFs of SARS-CoV-2 are also involved in the inflammatory mechanism of the infection process. ORF3d N-terminal and ORF8 proteins elicit strong antibody responses [32]. ORF9b also activates the inflammasome to evade the immune response and promote viral replication [48]. ORF3b [29], ORF6 [34,35], ORF7a [38], ORF8 [43,44], and ORF9c [31,49,50,51] of SARS-CoV are important interferon-I antagonists, which induce impaired host immune responses [138]. ORF7a hijacks the host ubiquitin system for enhancing the ability to antagonize interferon-I responses [39]. ORF8 interacts with the major histocompatibility complex (MHC-I) [24], activates the IL-17 signaling pathway, and promotes pro-inflammatory cytokines [45]. ORF9c protein interacts with the related proteins of NFκB-related molecules [31] and impairs antigen processing and presentation and complement signaling, and induces IL-6 signaling [49]. ORF7a binds to CD14^+^ monocytes, leading to a decrease in antigen presentation and inducing the marked expression of pro-inflammatory cytokines [40].

### 4.1. Mitochondria Induce NLRP-3-Based Inflammatory Response 

Pro-inflammatory NLRP-3 signaling activates cytokines, thus generating mitochondrion-related inflammatory cascades [150,151,152]. This is particularly concerning in elderly patients, who experience an increase in the NLRP-3 inflammasome with age [153]. This exacerbates SARS-CoV-2 infection [154]. The mitochondrial phospholipid cardiolipin can directly bind and activate the inflammasome complex NLRP3 [152]. Mitochondrial-generated ROS are involved in the activation of the NLRP3 inflammasome [152]. Stimulation of the NLRP3 inflammasome leads to the activation of caspase-1, which, in turn, leads to the proteolysis of cytokines such as interleukin (IL)-1β and IL-18 [54]. ORF3a activates pro-IL-1β gene expression and IL-1β secretion, which ultimately activates NFκB signaling and the NLRP3 inflammasome, promoting cytokine storm [27].

Viral proteins directly interact with components or regulatory proteins of NLRP3, including the interaction with the NLRP3 receptor via nucleocapsid protein, and the interaction of ORF3a with Caspase 1 and TNF receptor-associated factor 3 (TRAF3) [27,28,155]. In addition to the canonical NLRP3 activation pathways of PAMPs and DAMPs, the envelope proteins ORF3a and ORF8b of SARS-CoV act as NLRP3 agonists [27,156,157] and play a role in the inflammatory pathogenesis [158,159]. In addition, transcription of NFκB-dependent target genes of pro-inflammatory cytokines, such as IL-18 and IL-1b, is increased through nucleocapsid, spike, ORF7a, and OFR3a proteins [28,155,160,161]. ORF3a protein stimulates the NLRP3 cascade in bone marrow macrophages, and induces IL-1β-mediated inflammatory responses [151,162].

### 4.2. Activation of TLR7 and TLR9 Signaling

During SARS-CoV-2 infection, TLRs first bind their ligands and then recruit adapter proteins such as myeloid differentiation primary response 88 (MyD88) and adapter-inducible interferon-β (TRIF)-containing the TIR domain, which activate downstream immune molecular pathways [163], thus protecting the body from invading antigens. However, excessive activation of TLRs can lead to severe inflammatory responses, resulting in injury and even death [56,164]. Furthermore, when mtROS are increased, it leads to mitochondrial dysfunction and mtDNA leakage, which induces TLR9 and NFκB activation and the release of inflammatory cytokines [165].

TLR7 can be activated by ssRNAs rich in guanosine (G) and uridine (U) [166]. Two GU-rich ssRNA sequences in SARS-CoV-2 can activate TLR7 and its downstream MyD88-related pathway in human dendritic cells [167]. TLR7 recognizes SARS-CoV-2 in immune cells, triggering the production of type I interferon and pro-inflammatory cytokines [168,169]. TLR9 can recognize cytosine-guanine (CpG)-rich sequences in viral and mtDNA [170], and CpG in the SARS-CoV-2 envelope protein and ORF10 coding region can be recognized by TLR9 as a ligand [171]. TLR9 promotes inflammation through NFκB signaling and IL-6 production, and it impairs endothelial cell function by reducing endothelial nitric oxide synthase (eNOS) expression [165].

### 4.3. MAVS Signaling Regulates Inflammatory Responses

Following viral infection, MAVS initiates the innate immune response through the mitochondria. MAVS is mediated by the interaction of translocases with mitochondrial outer membrane proteins (TOM); for example, STING (MITA)—a transmembrane protein present on the endoplasmic reticulum, mitochondria, and mitochondria-associated membranes—contributes to the activation of MAVS [172]. Ring Finger Protein 5 (RNF5) regulates MAVS signaling by ubiquitinating MITA [173].

TOM70 is involved in protein transport for electron transport chain complex assembly in mitochondria [174]. TOM70 interacts with TANK-binding kinase (TBK1) through the heat shock protein 90 (HSP90), in order to bring the signal outside the mitochondria [175], thus activating the interferon response. TOM70 activates TBK1 through HSP90, taking the signal outside the mitochondria to activate interferon [50]. When the interferon regulatory factor (IRF)-3 binds to HSP90, it triggers the MAVS complex, E3 links tumor necrosis factor receptor-associated factors 3 and 6 (TRAF3 and TRAF6) to activate NFκB, and IRF provides antiviral protection [176]. Meanwhile, the membrane protein of SARS-CoV-2 inhibits the aggregation of TRAF3, TBK1, and IRF3 into the MAVS complex [177]. ORF9b inhibits the interaction between TOM70 and HSP90 and forms a complex with TOM70, thus affecting the mitochondrial electron transport chain [51], reducing the host immune response regulated by interferon I [47], and inducing apoptosis [50].

Furthermore, SARS-CoV-2 blocks interferon 1 and interferon 3 by interfering with the MAVS pathway [178]. The membrane protein inhibits the activation of interferon by down-regulating MAVS [177,178]. NSP13 inhibits TBK1 phosphorylation and blocks the downstream of the MAVS pathway [38]. ORF6 blocks the induction of interferon by MDA-5, MAVS, and TBK1 [179]. ORF3 induces ubiquitination of interferon-α receptor subunit 1 (IFNAR1) to down-regulate interferon 1 activity [180].

## 5. Therapeutic Strategies against COVID-19 Involving Mitochondria

**Figure 2:**
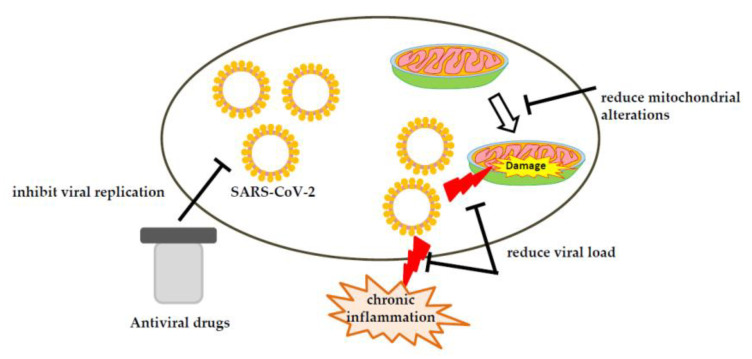
Using Therapeutic Strategies to Reduce Mitochondrial Disorder.

In people with long COVID and chronic fatigue syndrome, reducing the viral load and improving mitochondrial health may be beneficial (Figure 2). First, direct-acting antiviral drugs—especially analogs such as remdesivir—can effectively inhibit viral replication by blocking the activity of viral polymerase [181]. In addition to reducing viral load in cells, they may also reduce sources of chronic inflammation that lead to severe sepsis, multi-organ failure, and mitochondrial damage. However, the recent results of remdesivir treatment showed no benefit for long COVID-19 patients [182,183]. After one year of follow-up, one in six survivors considered recovery from COVID-19 incomplete, and one in four was troubled by fatigue [182]. Another study has reported that one-third of patients experienced fatigue 6 months after COVID-19 [183].

Generalized antioxidants, such as N-acetylcysteine [184], glutathione [185], and catalase [186], can effectively reduce mitochondrial changes, as they restore and protect mitochondrial function [187], thereby reducing the spread of the virus SARS-CoV-2. Mitochondrial oxidative stress can also be reduced using mitochondrion-targeted catalytic antioxidants such as MnTBAP [188], EUK-8, and EUK-134 [189], as well as Mito-TEMPO and mitoquinol [112,162,190]. In addition, mitochondrial quinone/mitochondrial phenol mesylate (Mito-MES) has been demonstrated to possess significant antiviral activity against SARS-CoV-2 and can reduce excessive inflammation in the host [191]. Effective treatment can also be obtained with mPTP inhibitors such as NIM811 [192,193].

IL-6R and IL-1 receptor blockers are useful in the treatment of cytokine release syndrome [194,195]. For example, the IL-6R-blocking antibody Tocilizumab can be used to treat severe cytokine release syndrome [196,197] and SARS-CoV-2 infection [198]. Likewise, the IL-1 receptor antagonist anakinra significantly improved survival in SARS-CoV-infected mice with over-active NLRP3 inflammasome [195,199,200]. Increased survival and clinical improvement were observed in patients with COVID-19 adult respiratory distress syndrome receiving high-dose intravenous anakinra [201].

In addition, SARS-CoV-2-infected cells mainly rely on glycolysis to meet their energy demands, due to mitochondrial respiratory dysfunction. Inhibition of glycolysis in infected cells has been found to inhibit viral proliferation [107]. Ruthenium red—a mitochondrial calcium uniporter (MCU) inhibitor—also normalizes mitochondrial morphology and function in HIV-infected cells [202]. Other known MCU inhibitors include ruthenium 265, mitoxantrone, and the antibiotic doxycycline [203]. All of these strategies may help to improve mitochondrial health in people with long COVID or chronic fatigue syndrome.

## 6. Conclusions

Managing long COVID is a major challenge, due to the lack of effective treatments at present. The current understanding of the pathophysiology of long COVID suggests that mitochondrial dysfunction may be one of the underlying mechanisms. Although the exact mechanism by which SARS-CoV-2 induces mitochondrial dysfunction is not fully understood, accumulating evidence has supported such a notion. Therefore, targeting mitochondrial function may represent a new therapeutic approach for long-COVID patients. Several compounds, including antioxidants, mitochondrion-targeting peptides, and modulators of mitochondrial biogenesis, have shown promise for the treatment of mitochondrial dysfunction, in pre-clinical studies. However, further studies are needed to determine the safety and efficacy of mitochondrial-targeted therapies in the context of long COVID.

## Figures and Tables

**Table 1:** Summary of non-structural proteins of SARS-CoV-2 and their functions.

Proteins	Length (AA) ^1^	Function, Key Features
NSP1	180	Suppresses host genes and enhances viral RNA expression
NSP2	638	For SARS-CoV replication
NSP3	1945	Component of the replication/transcription complex
NSP4	500	Assembly of cytoplasmic double-membrane vesicles required for viral replication
NSP5	306	Cleavage of viral polyproteins
NSP6	290	Inhibits the lysosomal autophagy system and stimulates the NLRP3 inflammasome-dependent pyroptotic pathway
NSP7	83	Involved in the replication and transcription of SARS
NSP8	198	Viral RNA primers co-localize with RdRP to replicate SARS-CoV
NSP9	113	Interact with the replication complex
NSP10	139	Interact with NSP14 and NSP16
NSP11	13	Unknown
NSP12	932	RNA-dependent RNA polymerase
NSP13	601	Zinc-binding domain, NTPase, dNTPase. 5′-to-3′ RNA and DNA helicase, RNA 5′-triphosphate
NSP14	527	3′-to-5′ exoribonuclease, zinc-binding domain, and N7-methyltransferase
NSP15	346	Uridylate-specific endoribonuclease, homohexamer
NSP16	298	Putative ribose-2′-O-methyltransferase

^1^ from SARS-CoV-2 reference genome (NC_045512.2). dNTPase, deoxynucleoside triphosphohydrolase; NLRP3, NLR family pyrin domain-containing 3; NSP, non-structural proteins; NTPase, nucleoside-triphosphatase; SARS-CoV, Severe acute respiratory syndrome-associated coronavirus.

**Table 2:** Summary of SARS-CoV-2 open reading frame proteins and their functions.

Proteins	Length (AA)	Function (Ref)
ORF3a	275	Ion channel [25] Replication and virulence [26] Activating pro-IL-1β gene expression and IL-1β secretion [27] Ultimately activating NFκB signaling and NLRP3 inflammasomes [27] Promoting the cytokine storm [27] Necrotic cell death and lysosomal damage [28]
ORF3b	22	IFN antagonism [29]
ORF3c	41	Unknown [30]
ORF3d	57	Interaction with STOML2 [31] With ORF8, elicits the strongest antibody responses [32]
ORF6	61	Localized to the ER, autophagosomes, and lysosomes [33] Blocks STAT transportation from the cytoplasm to the nucleus [34] IFN antagonist [34,35] Inhibits STAT1 nuclear transportation [36] Accumulation of hnRNPA1 in the nucleus [37]
ORF7a	121	Antagonizes the IFN-I response [38] Hijacks the host ubiquitin system to enhance and antagonize IFN-I responses [39] Decreases antigen-presenting ability and induces expression of pro-inflammatory cytokines [40]
ORF7b	43	Transmembrane protein localized in the Golgi [41]
ORF8	121	Apoptosis [42] Antagonizing the IFN signaling pathway [43] Interacts with MHC-I [24] IFN I antagonist [44] Activates IL-17 signaling pathway and promote proinflammatory factors [45]
ORF9b	97	Induces autophagy in host cells mediated by ATG5 [46] Tom70 forms a complex with ORF9b to modulate the host immune response by compromising type I IFN synthesis [47] Activation of inflammasome [48] Promotes proteasomal degradation of Drp1 [46]
ORF9c	73	Protein interaction between ORF9c protein and NFκB-related molecules [31] Interactions with NFκB-related molecules [49] Interferes with IFN signaling [31,50,51]
ORF 10	38	Unknown [52]

ATG5, autophagy-related 5; Drp1, dynein-related protein 1; ER, endoplasmic reticulum; hnRNPA1, heterogeneous nuclear ribonucleoprotein A1; IFN, interferons; IL, interleukin; MHC, major histocompatibility complex; NFκB, nuclear factor kappa-light-chain-enhancer of activated B cells; NLRP3, NLR family pyrin domain-containing 3; ORF, open reading frame; STAT1, signal transducer and activator of transcription 1; STOML2, stomatin-like 2.

## Data Availability

No new data were created or analyzed in this study. Data sharing does not apply to this article.
